# Supplementary material for: Cryo-EM observation of the amyloid key structure of polymorphic TDP-43 amyloid fibrils
Source: Nat Commun. 2024 Jan 12;15:486. doi: 10.1038/s41467-023-44489-0 (PMC10784485; doi:10.1038/s41467-023-44489-0)
Supplement: Supplementary file 1 — Supplementary information [file 41467_2023_44489_MOESM1_ESM.pdf]

Supplementary information

Cryo-EM observation of the amyloid key structure of polymorphic TDP-43 amyloid fibrils

Kartikay Sharma[1*], Fabian Stockert[1], Jayakrishna Shenoy[2], Mélanie Berbon[2], Muhammed Bilal Abdul-Shukkoor[2], Birgit Habenstein[2], Antoine Loquet[2], Matthias Schmidt[1], Marcus Fändrich[1]

Affiliations

[1] Institute of Protein Biochemistry, Ulm University, 89081 Ulm, Germany

[2] Univ. Bordeaux, CNRS, Bordeaux INP, CBMN, UMR 5248, IECB, Pessac, France

Correspondence

*Correspondence to: kartikay.sharma@uni-ulm.de

Supplementary figures

Supplementary Figure 1

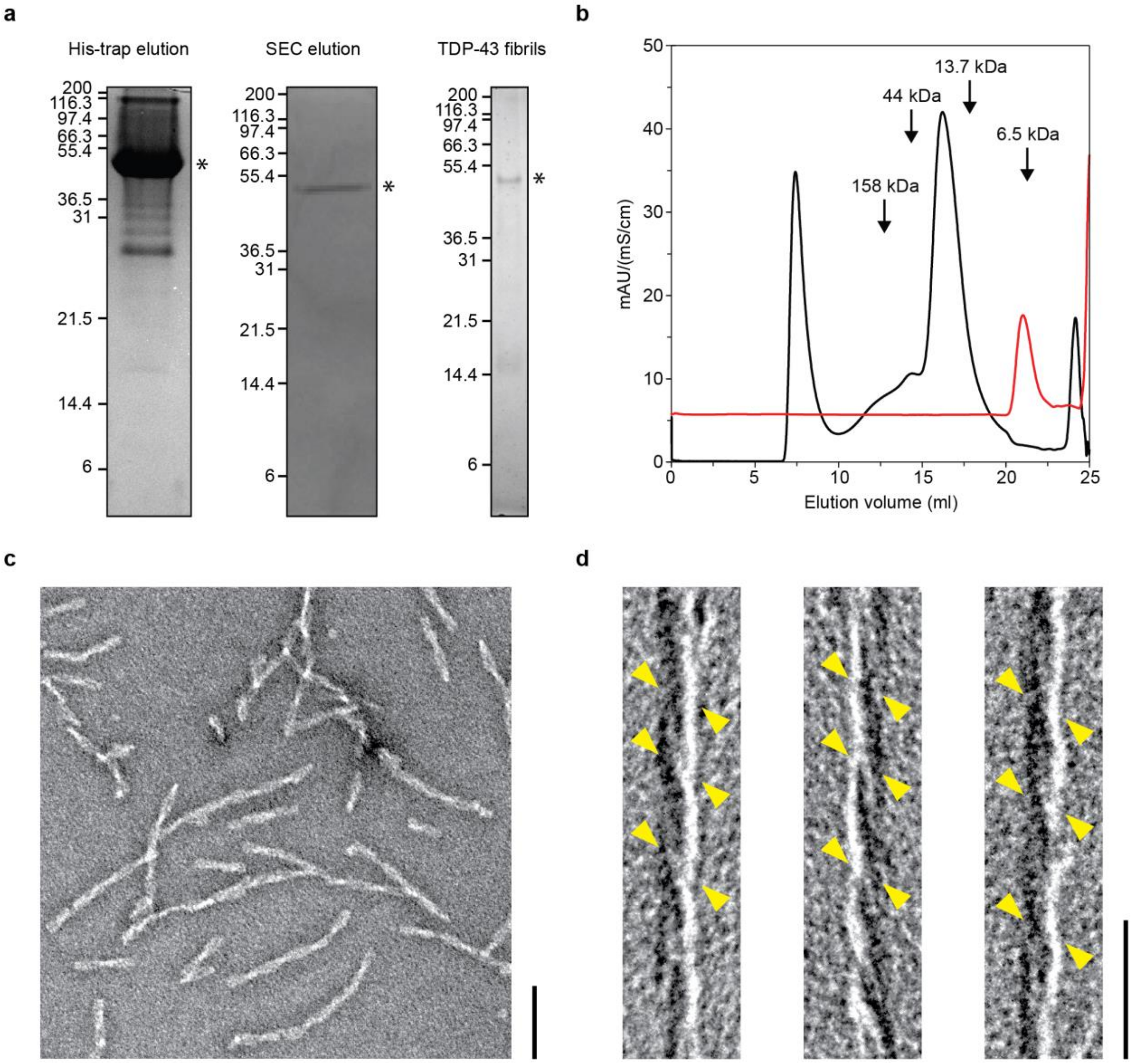

Supplementary Figure 1.

TDP-43 fibrils and their handedness.

(a) Representative images of denaturing protein gels for TDP-43 protein purification and fibril formation. Histrap elution (left), size exclusion chromatography (SEC) elution (middle), TDP-43 fibrils (right). The asterisks indicate the TDP-43 protein. (b) Chromatogram from SEC column. UV scale: black, conductivity scale: red (c) Negative-stain TEM image of the formed

fibrils. (d) TEM images of platinum side-shadowed TDP-43 fibrils. Scale bar: 50 nm. Arrow heads are drawn to guide the eye.

Supplementary Figure 2

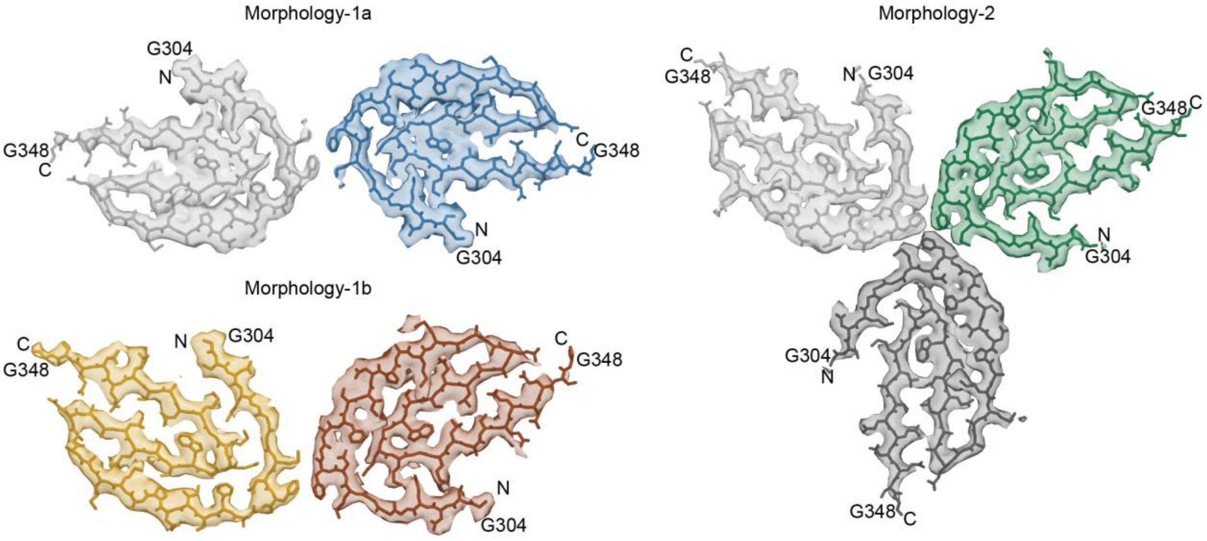

Supplementary Figure 2.

Cross-sectional views of the 3D maps and models.

Cross-sectional view of single molecular layers of the 3D maps (surface rendered, zoned around the atomic models), superimposed with the respective molecular model (stick representation).

Supplementary Figure 3

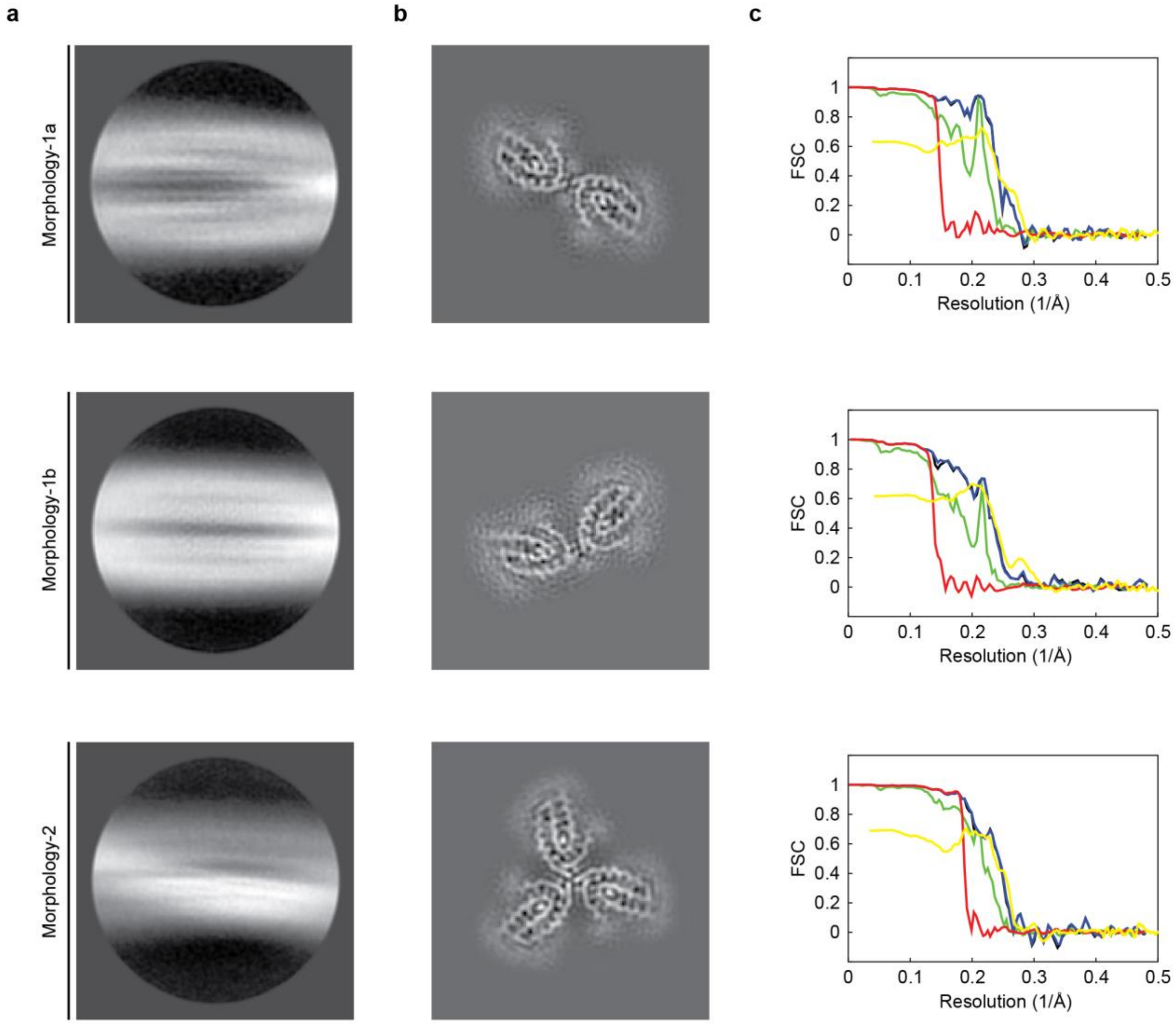

Supplementary Figure 3.

Cryo-EM data processing of fibril Morphologies-1a, 1b and 2.

(a) Representative 2D classes of fibril Morphologies-1a, 1b and 2. (b) 5.1 Å thick central slices of the 3D maps of Morphologies-1a, 1b and 2. (c) FSCs between the two half-maps and between refined atomic models and cryo-EM density maps of Morphologies-1a, 1b and 2. Black: FSC corrected, green: FSC unmasked, blue: FSC masked, red: corrected FSC phase randomized masked map, yellow: FSC map-model.

Supplementary Figure 4

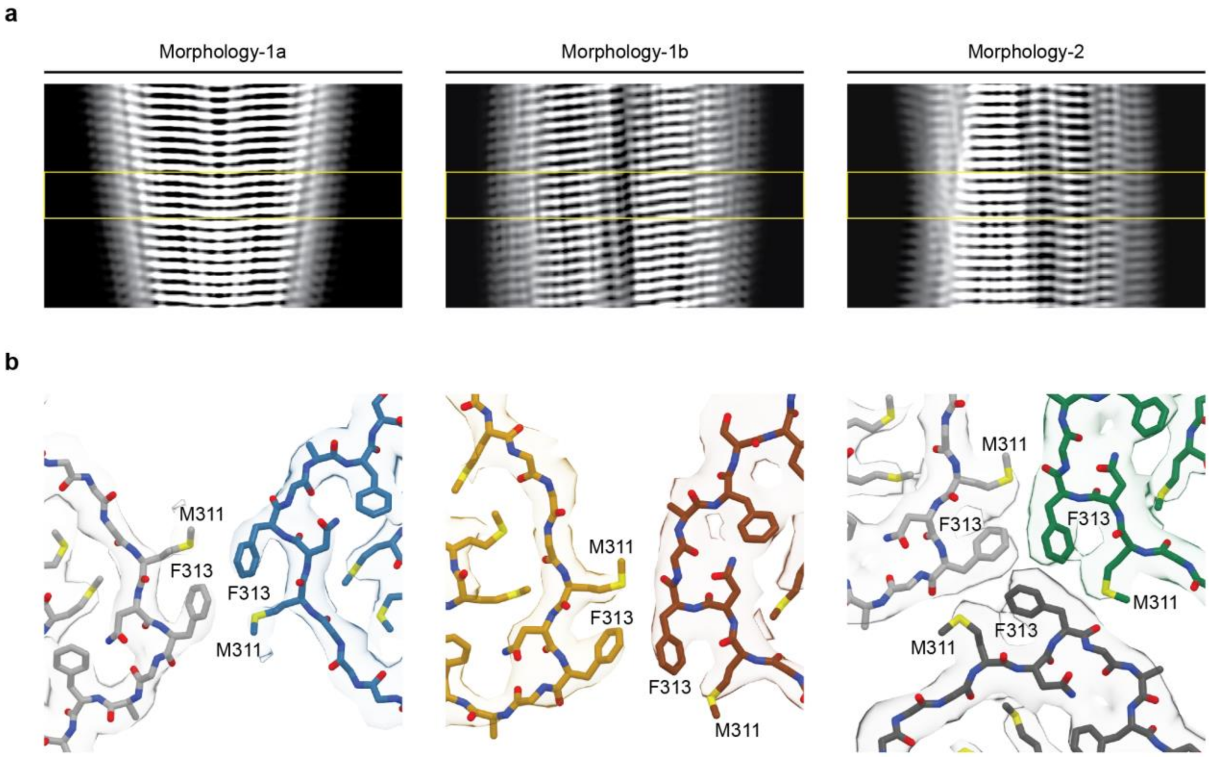

Supplementary Figure 4.

Fibril protein stacks and their interfaces in Morphologies-1a, 1b and 2.

(a) 2D projection of 3D maps of fibril Morphologies-1a, 1b and 2. Yellow lines are drawn to guide the eye. (b) Interfaces between the fibril protein stacks of Morphologies-1a, 1b and 2.

Supplementary Figure 5

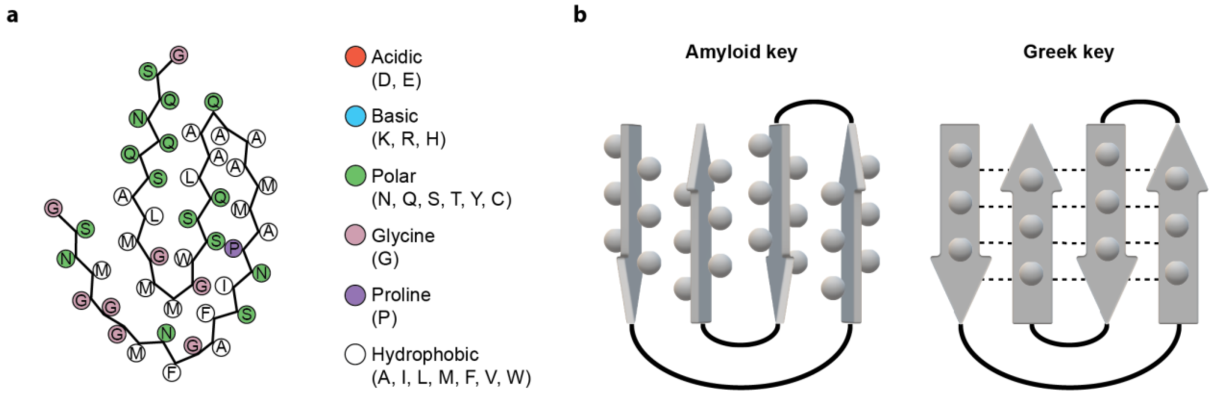

Supplementary Figure 5.

The amyloid key and the Greek key.

(a) Schematic representation of the fibril protein. (b) Schematic representation of the amyloid key and the Greek key. Arrows and spheres indicate the β-strands and the side chains, respectively. Continuous line represents the loops connecting the β-strands. The backbone hydrogen bonds are represented as dotted lines.

Supplementary Figure 6

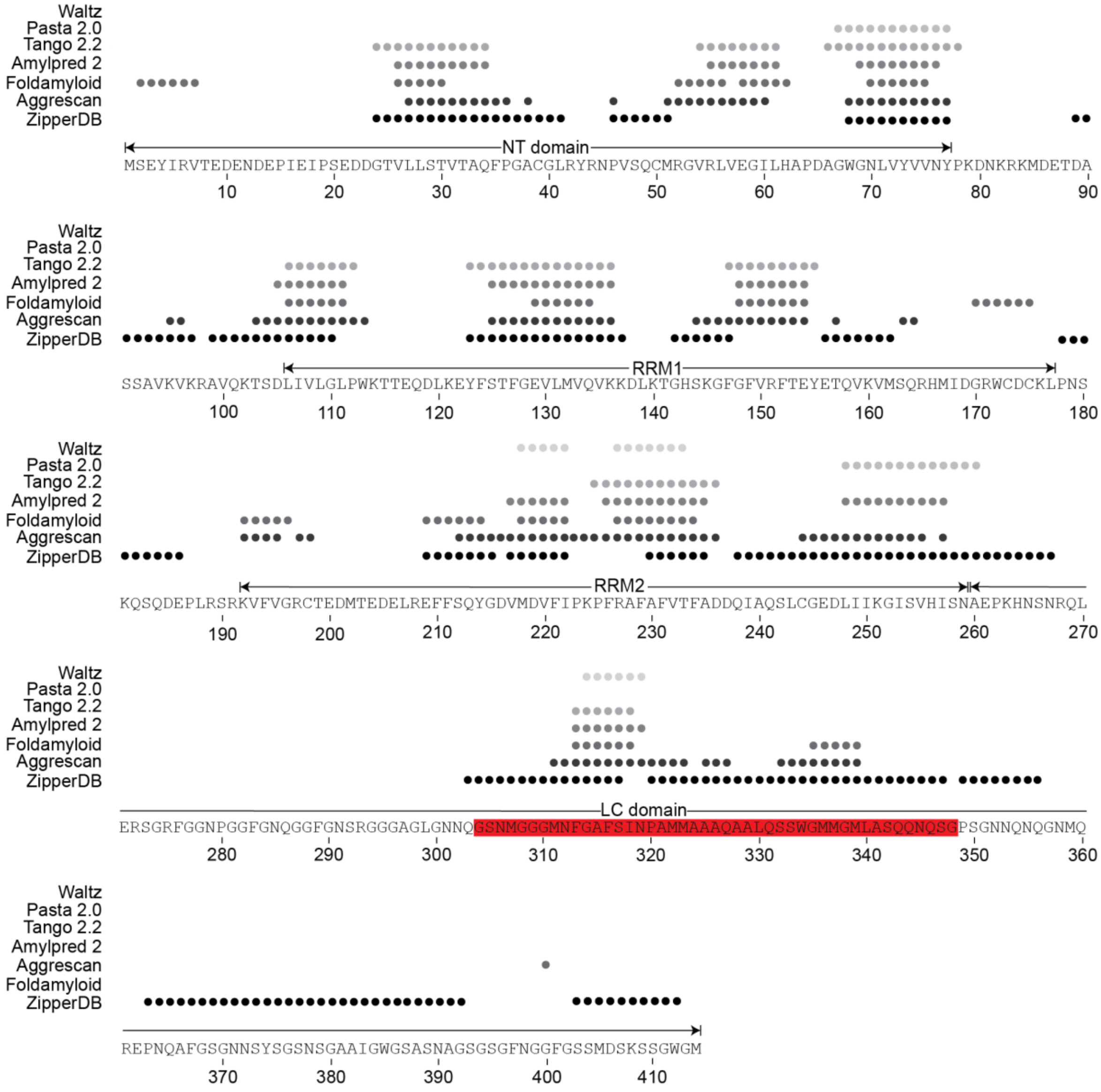

Supplementary Figure 6.

Aggregation prone regions of TDP-43 protein.

Aggregation prone residues (dots) of TDP-43 protein as predicted by seven different algorithms (ZipperDB, Aggrescan, Foldamyloid, Amylpred 2, TANGO 2.2, PASTA 2.0 and Waltz as indicated in the panel). Sequence of fibril protein segment is highlighted with a red background.

Supplementary Figure 7

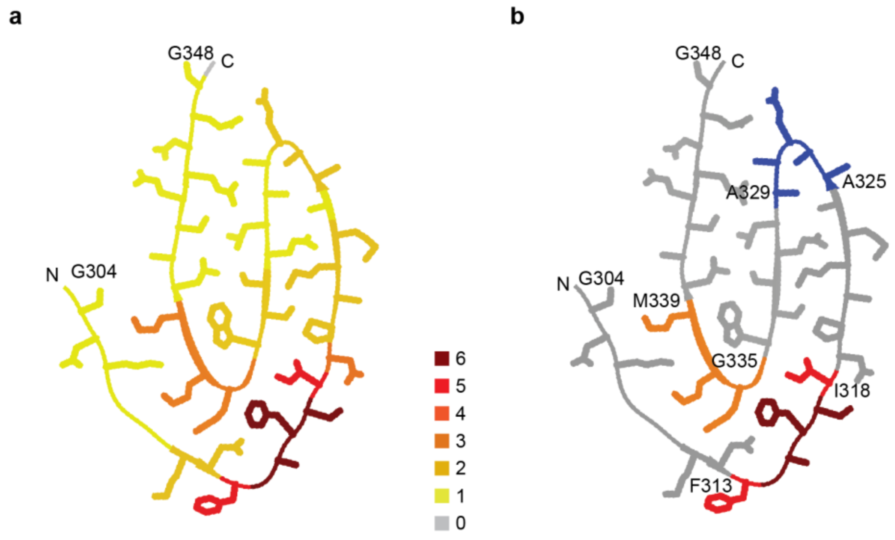

Supplementary Figure 7.

Location of the aggregation prone regions in the fibril core.

(a) Schematic representation of the fibril protein showing the location of the aggregation prone regions. Residues are color coded according to the aggregation score 0–6. (b) Definition of two most aggregation prone regions (residue Phe313 to Ile318, red, and Gly335 to Met339, orange) and of the arc region (residues Ala325 to Ala329, blue).

Supplementary Figure 8

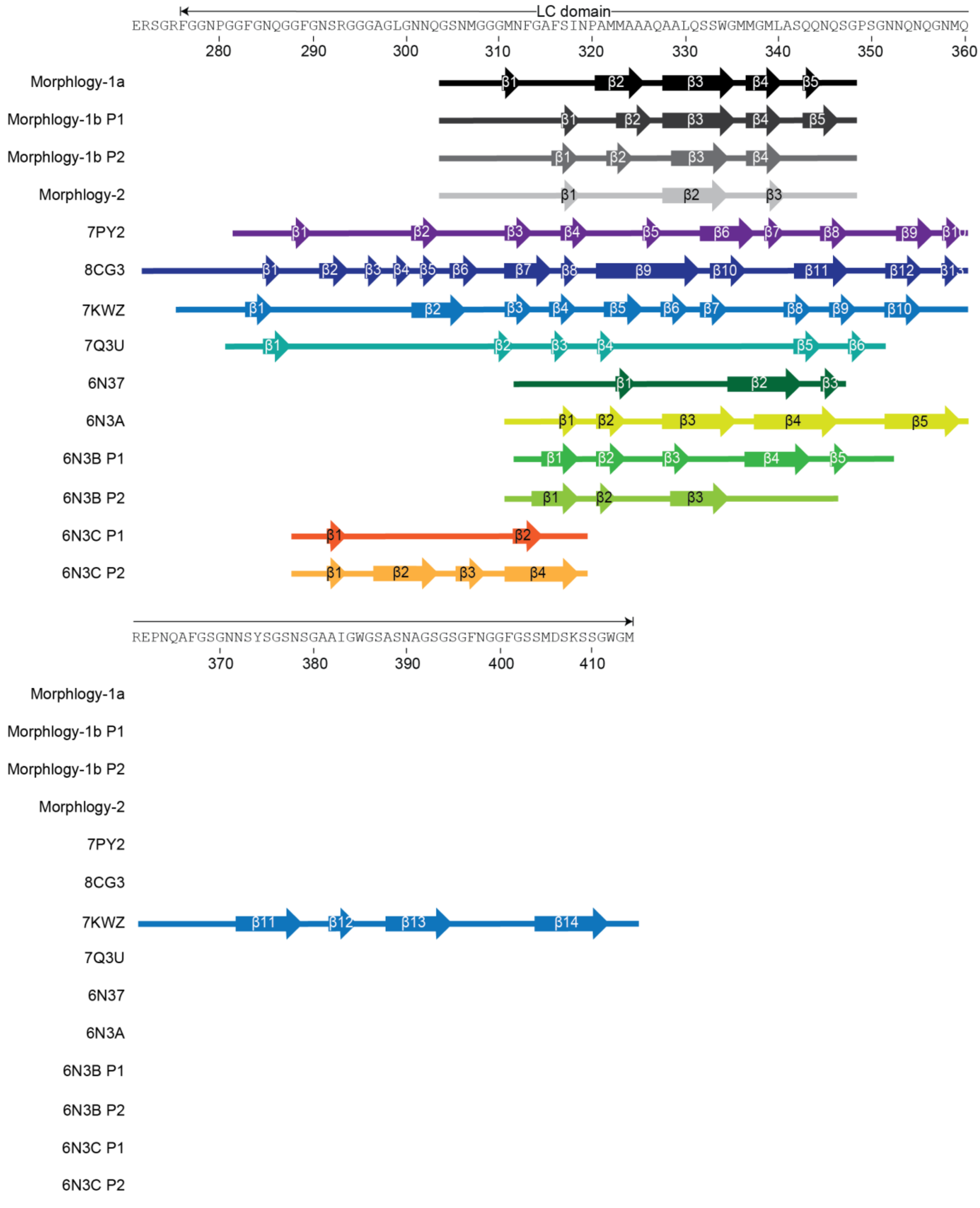

Supplementary Figure 8.

Comparison of the β-sheet structure in different TDP-43-derived fibrils.

Comparison of the fibril core of our structures and in previously described TDP-43-derived fibrils. Arrows indicate β-strands. PDB IDs are given for the published fibril structures.

Supplementary Figure 9

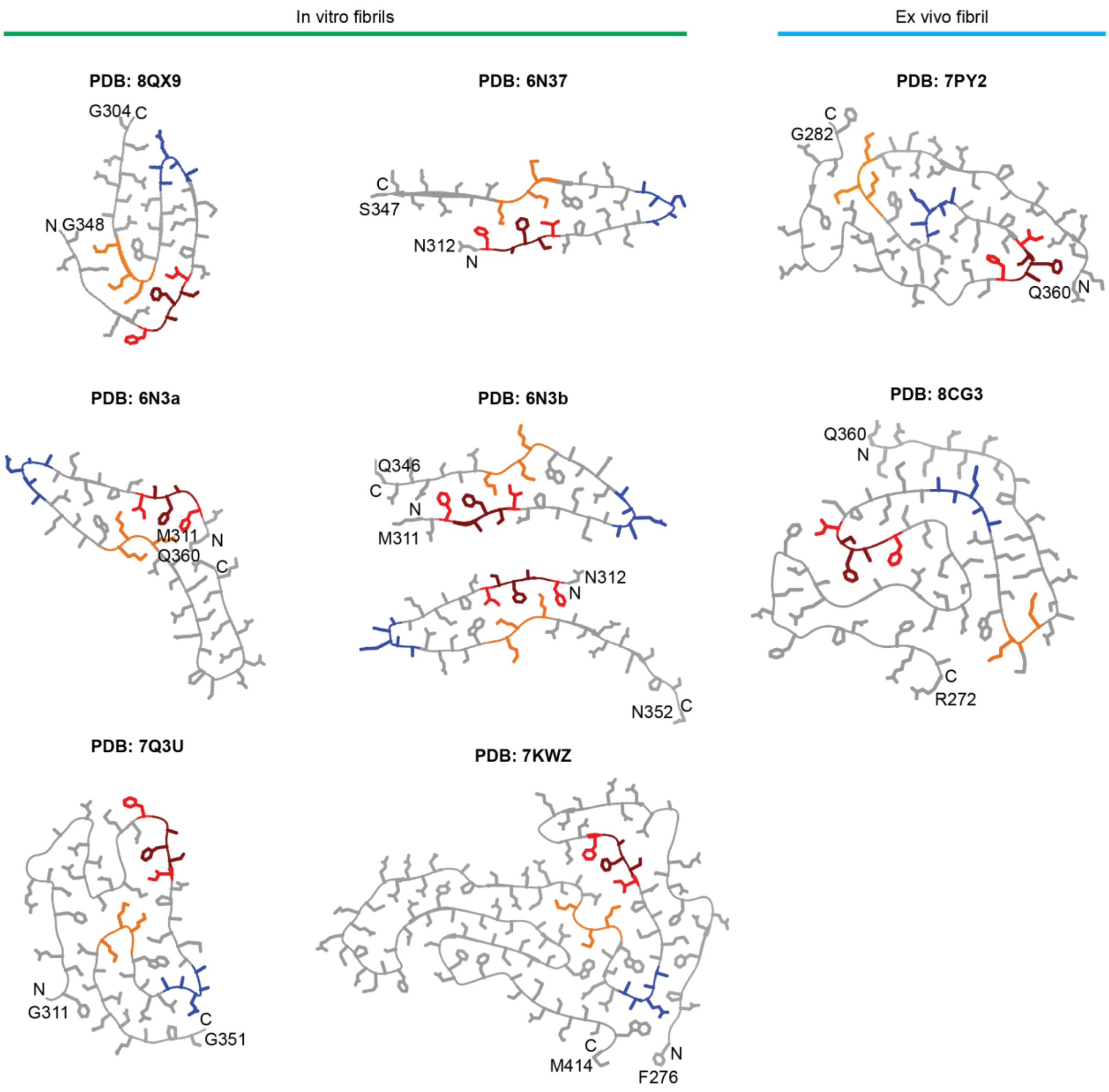

Supplementary Figure 9.

Comparison of the fibril protein fold in TDP-43-derived fibrils.

Comparison of the fibril protein folds of different TDP-43 fibrils with the most aggregation prone regions (residue Phe313 to Ile318 and residue Gly335 to Met339) colored in red and orange, and the arc of our structure labelled in blue (see Supplementary Fig. 5). In case of PDB entry 6N3b, two fibril protein stacks are shown: top: stack 1; bottom: stack 2.

Supplementary tables

Supplementary Table 1

**Data collection**			
**Magnification**	X 130,000		
**Voltage [kV]**	300		
**Total dose [eÅ^{-2}]**	52.08		
**Defocus Range [µm]**	-1.2 to -2.5		
**Pixel size [Å]**	1.04		
**Exposure time [s per frame]**	0.25		
**Number of frames**	40		
**Reconstruction**			
	**Morphology-1a**	**Morphology-1b**	**Morphology-2**
**Box size [pix]**	220	230	256
**Inter-box distance [Å]**	27.64	27.64	27.64
**Symmetry**	C2	C1	C3
**Micrographs**	1,641	1,641	1,641
**Initial number of particles**	11,560	26,881	22,126
**Final number of particles**	11,560	26,881	14,995
**Map resolution,**<br>**0.143 FSC criterion [Å]**	3.76	4.05	3.86
**B-factor [Å^2]**	-105.53	-136.51	-111.28
**Rise [Å^2]**	4.78	4.79	4.77
**Twist [°]**	-1.77	-1.8	-1.3

Supplementary Table 1.

Statistics of the cryo-EM data collection and image processing.

Supplementary Table 2

	**Morphology-1a**	**Morphology-1b**	**Morphology-2**
**Initial model used**	De novo	De novo	De novo
**Map resolution, 0.143 FSC criterion [Å]**	3.76	4.05	3.86
**Model composition**			
**Chains**	12	12	18
**Non-hydrogen atoms**	7,164	7,164	10,746
**Protein residues**	540	540	810
	0	0	0
**Ligands**	0	0	0
**Root mean square deviations**			
**Bond length [Å]**	0.013	0.013	0.013
**Bond angles [Å]**	2.304	2.276	2.382
**Validation**			
**Molprobity[1] score**	1.14	1.43	1.00
**Clash score**	2.8	4.19	1.77
**Poor rotamers [%]**	0	0	0
**Cβ outliers [%]**	0	0	0
**Ramachandran plot**			
**Favored [%]**	97.67	96.51	97.67
**Allowed [%]**	2.33	3.49	2.33
**Disallowed [%]**	0	0	0
**B-factor [$Å^2$]**	-105.53	-136.51	-111.28
**Model resolution [Å]**	3.58	3.86	3.78

Supplementary Table 2.

Structural statistics of model building and refinement.

Supplementary references

1. Chen, V. B. *et al.* MolProbity: all-atom structure validation for macromolecular crystallography. *Acta Crystallogr D Biol Crystallogr* **66**, 12–21 (2010).
