# Supplementary material for: Cryo-EM observation of the amyloid key structure of polymorphic TDP-43 amyloid fibrils
Source: Nat Commun. 2024 Jan 12;15:486. doi: 10.1038/s41467-023-44489-0 (PMC10784485; doi:10.1038/s41467-023-44489-0)
Supplement: Supplementary file 3 — Reporting Summary [file 41467_2023_44489_MOESM3_ESM.pdf]

nature portfolio

Corresponding author(s): Kartikay Sharma

Last updated by author(s): 04.12.2023

# Reporting Summary

Nature Portfolio wishes to improve the reproducibility of the work that we publish. This form provides structure for consistency and transparency in reporting. For further information on Nature Portfolio policies, see our Editorial Policies and the Editorial Policy Checklist.

## Statistics

For all statistical analyses, confirm that the following items are present in the figure legend, table legend, main text, or Methods section.

| n/a | Confirmed | |
|---|---|---|
| ☐ | ☒ | The exact sample size (*n*) for each experimental group/condition, given as a discrete number and unit of measurement |
| ☐ | ☒ | A statement on whether measurements were taken from distinct samples or whether the same sample was measured repeatedly |
| ☒ | ☐ | The statistical test(s) used AND whether they are one- or two-sided<br>*Only common tests should be described solely by name; describe more complex techniques in the Methods section.* |
| ☒ | ☐ | A description of all covariates tested |
| ☒ | ☐ | A description of any assumptions or corrections, such as tests of normality and adjustment for multiple comparisons |
| ☐ | ☒ | A full description of the statistical parameters including central tendency (e.g. means) or other basic estimates (e.g. regression coefficient) AND variation (e.g. standard deviation) or associated estimates of uncertainty (e.g. confidence intervals) |
| ☒ | ☐ | For null hypothesis testing, the test statistic (e.g. *F*, *t*, *r*) with confidence intervals, effect sizes, degrees of freedom and *P* value noted<br>*Give P values as exact values whenever suitable.* |
| ☒ | ☐ | For Bayesian analysis, information on the choice of priors and Markov chain Monte Carlo settings |
| ☒ | ☐ | For hierarchical and complex designs, identification of the appropriate level for tests and full reporting of outcomes |
| ☒ | ☐ | Estimates of effect sizes (e.g. Cohen's *d*, Pearson's *r*), indicating how they were calculated |

*Our web collection on statistics for biologists contains articles on many of the points above.*

## Software and code

Policy information about availability of computer code

| | |
|---|---|
| Data collection | SerialEM v3.9.0 |
| Data analysis | RELION v3.1.1, MotionCor2, IMOD v4.9.0, CTFFIND v4.1, Coot v0.9.1, Phenix (Molprobity) v1.20.1-4459, Fiji (ImageJ) v1.53, UCSF Chimera 1.15, Situs (pdbsymm) 3.1 |

For manuscripts utilizing custom algorithms or software that are central to the research but not yet described in published literature, software must be made available to editors and reviewers. We strongly encourage code deposition in a community repository (e.g. GitHub). See the Nature Portfolio guidelines for submitting code & software for further information.

## Data

Policy information about availability of data

All manuscripts must include a data availability statement. This statement should provide the following information, where applicable:

- Accession codes, unique identifiers, or web links for publicly available datasets
- A description of any restrictions on data availability
- For clinical datasets or third party data, please ensure that the statement adheres to our policy

Cryo-EM datasets were deposited in the Electron Microscopy Public Image Archive under accession codes EMPIAR-11745. The reconstructed cryo-EM maps were deposited in the Electron Microscopy Data Bank with the accession codes: Morphology-1a: EMD-18715 [https://www.ebi.ac.uk/pdbe/entry/emdb/EMD-18715]; Morphology-1b: EMD-18716 [https://www.ebi.ac.uk/pdbe/entry/emdb/EMD-18716]; Morphology-2: EMD-18717 [https://www.ebi.ac.uk/pdbe/entry/emdb/EMD-18717]. The coordinates of the fitted atomic model were deposited in the Protein data bank (PDB) under the accession codes: Morphology-1a: 8QX9 [https://

doi.org/10.2210/pdb8qx9/pdb]; Morphology-1b: 8QXA [https://doi.org/10.2210/pdb8qxa/pdb]; Morphology-2: 8QXB [https://doi.org/10.2210/pdb8qxb/pdb]. The following previously published coordinates were used in Supplementary Fig. 7 and Supplementary Fig. 8: PDB entries: 6N37 [https://doi.org/10.2210/pdb6n37/pdb], 6N3A [https://doi.org/10.2210/pdb6n3a/pdb], 6N3B [https://doi.org/10.2210/pdb6n3b/pdb], 6N3C [https://doi.org/10.2210/pdb6n3c/pdb], 7Q3U [https://doi.org/10.2210/pdb7q3u/pdb], 7KWZ [https://doi.org/10.2210/pdb7kwz/pdb], 7PY2 [https://doi.org/10.2210/pdb7py2/pdb] and 8CG3 [https://doi.org/10.2210/pdb8cg3/pdb]. The data that support the findings of this study are available from the corresponding author upon reasonable request.

## Research involving human participants, their data, or biological material

Policy information about studies with human participants or human data. See also policy information about sex, gender (identity/presentation), and sexual orientation and race, ethnicity and racism.

| | |
|---|---|
| Reporting on sex and gender | Does not apply. |
| Reporting on race, ethnicity, or other socially relevant groupings | Does not apply. |
| Population characteristics | Does not apply. |
| Recruitment | Does not apply. |
| Ethics oversight | Does not apply. |

Note that full information on the approval of the study protocol must also be provided in the manuscript.

# Field-specific reporting

Please select the one below that is the best fit for your research. If you are not sure, read the appropriate sections before making your selection.

- [x] Life sciences
- [ ] Behavioural & social sciences
- [ ] Ecological, evolutionary & environmental sciences

For a reference copy of the document with all sections, see nature.com/documents/nr-reporting-summary-flat.pdf

# Life sciences study design

All studies must disclose on these points even when the disclosure is negative.

| | |
|---|---|
| Sample size | 1,641 cryo-EM images were collected. The sample size was not predetermined and image data collected based on the visibility of the fibrils. These data sets were used to extract 11,560 (Morphology-1a), 26,881 (Morphology-1b) and 22,126 (Morphology-2) particles for reconstruction based on the visibility of fibrils. |
| Data exclusions | 0 (Morphology-1a), 0 (Morphology-1b) and 7,131 (Morphology-2) particles were excluded during 3D classification steps. Classes of particles with low resolution were excluded. |
| Replication | EM data is based on a single sample. 11,560 (Morphology-1a), 26,881 (Morphology-1b) and 14,995 (Morphology-2) particles were used for the reconstruction. The measurement of the cross-over distance and the width were successfully replicated 50 times for each morphology. Fibril handedness was assessed by 3 technical replicates. All the replication were successfully shown same fibril handedness. |
| Randomization | The data shown represents a single case study, therefore randomization was not relevant for the purpose of this study. |
| Blinding | The data shown represents a single case study, therefore blinding was not relevant for this study. |

# Reporting for specific materials, systems and methods

We require information from authors about some types of materials, experimental systems and methods used in many studies. Here, indicate whether each material, system or method listed is relevant to your study. If you are not sure if a list item applies to your research, read the appropriate section before selecting a response.

## Materials & experimental systems

| n/a | Involved in the study | |
|---|---|---|
| x | | Antibodies |
| x | | Eukaryotic cell lines |
| x | | Palaeontology and archaeology |
| x | | Animals and other organisms |
| x | | Clinical data |
| x | | Dual use research of concern |
| x | | Plants |

## Methods

| n/a | Involved in the study | |
|---|---|---|
| x | | ChIP-seq |
| x | | Flow cytometry |
| x | | MRI-based neuroimaging |
